# A Fatigue Life Prediction Method Based on Strain Intensity Factor

**DOI:** 10.3390/ma10070689

**Published:** 2017-06-22

**Authors:** Wei Zhang, Huili Liu, Qiang Wang, Jingjing He

**Affiliations:** School of Reliability and Systems Engineering, Beihang University, Beijing 100191, China; zhangwei.dse@buaa.edu.cn (W.Z.); hlliu1993@163.com (H.L.); wangqiang@buaa.edu.cn (Q.W.)

**Keywords:** crack growth, fatigue life, fully reversed, nonlinear, strain intensity factor

## Abstract

In this paper, a strain-intensity-factor-based method is proposed to calculate the fatigue crack growth under the fully reversed loading condition. A theoretical analysis is conducted in detail to demonstrate that the strain intensity factor is likely to be a better driving parameter correlated with the fatigue crack growth rate than the stress intensity factor (SIF), especially for some metallic materials (such as 316 austenitic stainless steel) in the low cycle fatigue region with negative stress ratios R (typically R = −1). For fully reversed cyclic loading, the constitutive relation between stress and strain should follow the cyclic stress-strain curve rather than the monotonic one (it is a nonlinear function even within the elastic region). Based on that, a transformation algorithm between the SIF and the strain intensity factor is developed, and the fatigue crack growth rate testing data of 316 austenitic stainless steel and AZ31 magnesium alloy are employed to validate the proposed model. It is clearly observed that the scatter band width of crack growth rate vs. strain intensity factor is narrower than that vs. the SIF for different load ranges (which indicates that the strain intensity factor is a better parameter than the stress intensity factor under the fully reversed load condition). It is also shown that the crack growth rate is not uniquely determined by the SIF range even under the same R, but is also influenced by the maximum loading. Additionally, the fatigue life data (strain-life curve) of smooth cylindrical specimens are also used for further comparison, where a modified Paris equation and the equivalent initial flaw size (EIFS) are involved. The results of the proposed method have a better agreement with the experimental data compared to the stress intensity factor based method. Overall, the strain intensity factor method shows a fairly good ability in calculating the fatigue crack propagation, especially for the fully reversed cyclic loading condition.

## 1. Introduction

The fatigue life prediction is of great significance to the safety and reliability of structural components in many engineering projects. Numerous methods have been proposed for the fatigue life prediction [1,2]. Since the damage tolerance theory is widely accepted and adopted, the fatigue crack growth method (which is based on Linear Elastic Fracture Mechanics (LEFM)) is becoming increasingly important [3,4,5]. In 1963, Paris and Erdogan correlated the crack growth rate with the range of SIF and proposed the famous Paris equation, which is generally used for the fatigue life prediction of engineering structural materials [6,7,8]. The major issue is that the Paris equation does not account for the stress ratio effect. Thus, many modifications are proposed to account for additional factors [9,10,11]. For example, the fracture toughness and the stress ratio can be directly included as the modification coefficients in Forman’s equation [12,13], which is supposed to be a feasible approach to deal with the experimental data of many materials (especially for the high hardness alloys). Walker’s equation is also a general mathematical model with consideration of peak loading [10,14]. Elber proposes the effective SIF to modify the SIF range based on the crack closure concept [15]. Correia et al. propose a modified Castillo-Canteli-Siegele (CCS) model to calculate the crack growth rate (considering the plasticity-induced crack closure effect [4]). However, some experimental investigations indicate the insufficiency and even ineffectiveness of the crack closure [16,17]. Kujawski also advocates that the range of SIF is not the unique driving parameter of the crack growth rate, where the corresponding maximum load should be considered for some cases [18]. Some researchers propose local plastic characteristics (such as the plastic CTOD range and the plastic flow intensity factor) to correlate with the crack growth rate [19,20]. These driving parameters can describe the plastic deformation near the crack tip better than the SIF, especially under the “elastic-plastic” condition. However, their calculations usually involve the complex finite element analysis, which makes them inconvenient to use.

In most existing models [7,8,9,21,22,23], the SIF is usually considered as the appropriate driving parameter to correlate with the crack propagation rate. However, for some materials the strain range has shown a good ability to describe the fatigue crack growth in several studies [24,25,26,27,28,29]. Tomkins [30] proposes that the shear-off decohesion at the crack tip is the main driving factor for the crack growth. Chakrabortty [31] links the fatigue crack propagation with the cyclic plastic strain at the crack tip. Some experimental investigations reveal that the strain range can describe the fatigue life much better than the stress range for some materials (such as the 316 austenitic stainless steel [32,33]). These researches show that the cyclic plastic strain of stainless steel and other similar materials cannot be ignored during the fatigue tests [34,35,36]. For these types of the materials, the relationship between cyclic stress and strain is non-linear under the fully reversed applied loading, and even much smaller than the 0.2% proof strength. Several studies have found that compared with the SIF, the strain intensity factor could be a more effective parameter to describe the crack growth process, especially in the low fatigue cycle region for the R = −1 condition [37,38].

Usually, the applied loading is more available than the strain range in most practical cases. Moreover, in the current studies the strain intensity factor is directly calculated by using the experimental measurements of strain range rather than through the theoretical calculation based on the stress-strain analysis. In other words, an appropriate transformation algorithm between the SIF and strain intensity factor is necessary. In this paper, the application of the strain intensity factor for some materials under the fully reversed loading condition is discussed, and the corresponding transformation equation based on the cyclic stress-strain constitutive relationship is proposed. Then, the validation and comparison study is performed between the strain intensity factor and the SIF using the fatigue life testing data of smooth cylindrical specimens in 316 stainless steel and AZ31 magnesium alloy.

The paper is organized as follows: First, the strain intensity factor concept is introduced briefly; Second, the transformation relationship between the SIF and strain intensity factor is delivered, and a theoretical analysis is given to demonstrate the effectiveness of the proposed model; Third, the fatigue life prediction method based on strain intensity factor and modified EIFS is addressed; Fourth, the proposed model is validated by using the fatigue testing data in 316 austenitic stainless steel and AZ31 magnesium alloy; And finally, some conclusions are given.

## 2. Methodology

### 2.1. The Basic Concept of the Strain Intensity Factor

Irwin introduced the stress intensity factor Δ*K* as a driving parameter of the crack propagation (based on the small yield assumption [39]), while the SIF is defined as

(1) $$\Delta K=\gamma \Delta \sigma \sqrt{\pi a}$$

where Δ*K* represents the range of the stress intensity factor, *γ* represents the geometrical coefficient, *a* is the crack flaw size, and Δσ is the stress range. The SIF is the function of the global stress and geometry of the specimen, which can describe the local stress field near the crack tip. So, the SIF becomes a major driving parameter to correlate with the crack growth rate by many researchers [21,22,23]. However, some studies reveal that the strain intensity factor shows a better correlation with the crack growth for some materials under the fully reversed loading condition [37]. In those experimental studies, the SIF cannot give a satisfactory performance. Therefore, a strain-intensity-factor-based method is proposed in this paper. Similar to the SIF, for the simplest case (mode–I) the strain intensity factor is defined as follows:

(2) $$\Delta K_{\varepsilon }=\gamma \Delta \varepsilon \sqrt{\pi a}$$

where $\Delta K_{\varepsilon }$ is the range of the strain intensity factor and Δε is the strain range. 

**Figure 1:**
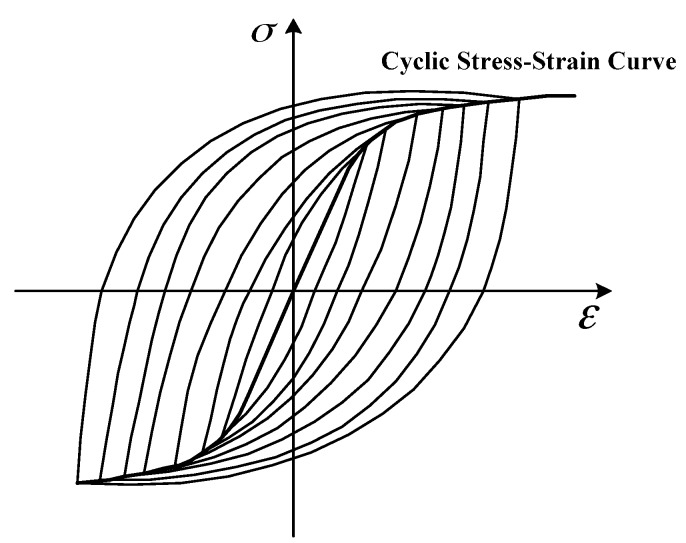
Cyclic stress-strain curve under fully reversed loading.

For the cyclic tension-tension load condition, the stress range and strain range vary linearly within the elastic region. For uniaxial, their relationship follows Hooke’s law, so the stress intensity factor and strain intensity factor are proportional. Therefore, the fatigue life predicted by the strain intensity factor should be the same as that obtained by using the SIF. However, for some materials under the tension-compression load condition, their cyclic stress and strain are not linearly related. The constitutive function should follow the cyclic stress-strain curve rather than the monotonic one (as shown in Figure 1). It can be seen that increasing the applied loading range will give rise to a more severe nonlinearity, and the hysteresis loop range will become wider. The formula between the cyclic stress and strain proposed by Ramberg-Osgood is expressed as Equation (3) [40]

(3) $$\varepsilon =\varepsilon_{e}+\varepsilon_{p}=\frac{\sigma }{E}+{\left(\frac{\sigma }{K^{\prime }}\right)}^{\frac{1}{n^{\prime }}}=f\left(\sigma \right)$$

where ε is the total strain, $\varepsilon_{e}$ is the elastic strain, $\varepsilon_{p}$ is the plastic strain, *K*′ represents the cyclic strength coefficient, and *n*′ represents the cyclic strain hardening coefficient.

**Figure 2:**
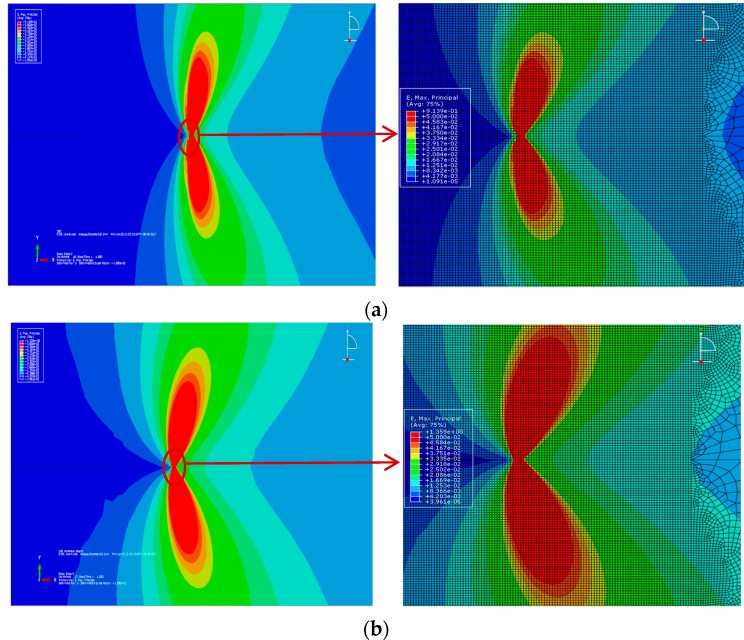
Strain field at the crack tip: (**a**) Linear elastic and perfect plastic; and (**b**) Nonlinear cyclic stress-strain relationship.

It is known that the flow bands at the crack tip will be activated under the cyclic tension-compression loading, which are induced by the dislocation movements. Crack propagation occurs when the accumulated cyclic strain exceeds the fracture strain [31]. So, the crack growth is more directly linked to the strain than to the stress. A 3D finite element simulation is conducted for a comparative demonstration. With the same geometry configuration and the applied stress range, different constitutive relationships will lead to a deviation of the global strain variation. Furthermore, the local strain fields at the crack tip are also quite different, which will ultimately influence the fatigue crack growth process. In Figure 2, the difference of the strain fields at the crack tip is visualized by using the finite element (FE) software ABAQUS. In Figure 2a,b the constitutive relationships follow the linear elastic and perfect plastic, and nonlinear cyclic stress-strain relationships respectively, which have an identical yield stress or 0.2% proof strength. The mechanical properties of the materials in the FE analysis are listed in Table 1.

The 0.2% proof strength of the material is 297 MPa; Young’s modulus and Poisson’s ratio are 202.5 GPa and 0.3 respectively. The cyclic strength coefficient and cyclic strain hardening coefficient are 691 and 0.154, while the applied loading is 200 MPa.

The simulation results of the remote strain and maximum local strain at the crack tip (for these two constitutive equations) are listed in Table 2. As seen in Table 2, under the same applied loading, the remote strain calculated by the nonlinear cyclic stress-strain relationship is larger than the linear elastic and perfect plastic one by 25.2%. The relative difference of the maximum local strain at the crack tip is 31.9%. It indicates that for some materials the strain range is a better parameter to describe the crack growth than the stress range.

Thus the relationship between the SIF range and the strain intensity factor range is also nonlinear in this condition. According to Equations (2) and (3), the formulation of the strain intensity factor under the fully reversed loading condition is proposed, which can be expressed as:

(4) $$\Delta K_{\varepsilon }=f^{-1}\left(\Delta \sigma \right)\gamma \sqrt{\pi a}=\frac{f^{-1}\left(\Delta \sigma \right)}{\Delta \sigma }\Delta K$$

**Figure 3:**
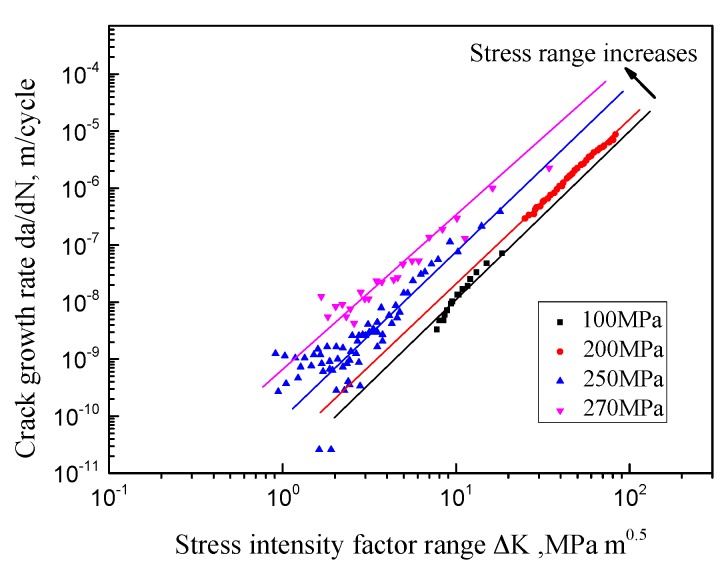
Crack growth rate vs. stress intensity factor (SIF) of 316 stainless steel.

To illustrate the difference between the SIF and the strain intensity factor in the correlation with the crack growth rate, the crack growth data in 316 stainless steel (under the fully reversed loading with different stress ranges) is analyzed. As shown in Figure 3, the lateral horizontal axis represents the SIF range and the vertical axis represents the crack growth rate. The dots in different shapes and colors represent the experimental measurements under different stress ranges. And all the data are from the fully reversed fatigue testing of cylindrical specimens in 316 stainless steel. Based on the traditional SIF method, all the testing data should shrink into one straight line, because of the same R-ratio. However, the testing data of different stress ranges follow several parallel straight lines. As shown in Figure 3, the lines in different colors represent the trends of the testing data under different stress levels. Distinct deviations are observed between the different groups of these data. Moreover, fixing the SIF range, the crack growth rate becomes larger with the stress range increasing. In this case, the SIF range is not the unique driving parameter for the crack growth rate.

**Figure 4:**
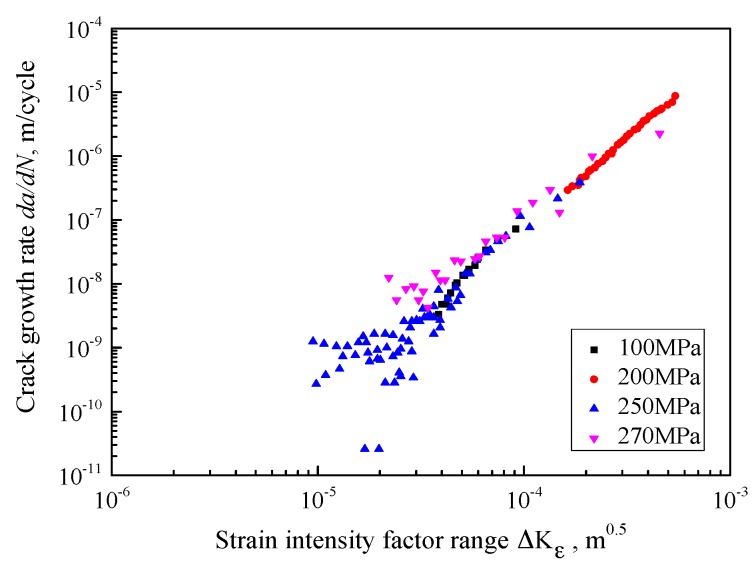
Crack growth rate vs. strain intensity factor range.

Using Equation (4), the strain intensity factor range $\Delta K_{\varepsilon }$ can be calculated, and the corresponding material properties are *n*′ = 0.154 and *k*′ = 691 [41]. The testing data are re-plotted in Figure 4. The horizontal axis represents the strain intensity factor range and the vertical axis represents the crack growth rate. It can be seen clearly that all the testing data shrink into one line (and the dispersion is quite small). It is indicated that the strain intensity factor range could be a better driving force than the SIF range to depict the fatigue crack propagation behavior. 

### 2.2. The Fatigue Life Prediction Model

**Figure 5:**
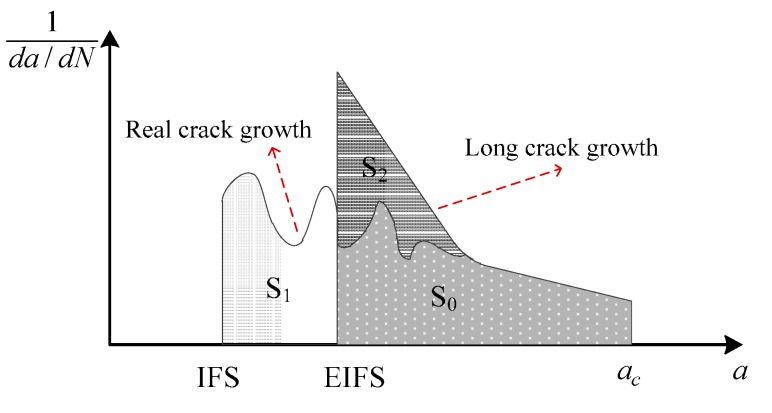
Schematic illustration of EIFS.

A SIF-based equation proposed by Newman is employed, shown as Equation (5) [42],

(5) dadN=C1[(1−g1−R)ΔK]n1(1−ΔKthΔK)p1(1−KmaxKc)q

where *C*_1_, *n*_1_, *p*_1_, and *q* are fitting parameters and *g* is related to the crack closure. *R* is the stress ratio. $\Delta K_{th}$, $K_{\max}$, and $K_{c}$ are the threshold SIF, the maximum SIF, and the critical SIF, respectively. The fast growing stage has little impact on the fatigue life and so can be ignored. Moreover, in this investigation R is equal to −1 (the fully reversed loading), so the Equation (5) can be simplified as:

(6) $$\frac{da}{dN}=C_{2}\Delta K^{n_{2}}{\left(1-\frac{\Delta K_{th}}{\Delta K}\right)}^{p_{2}}$$

where *C*_2_, *n*_2_, *p*_2_ are the fitting parameters. Next, the strain intensity factor replaces the SIF. The formula can be modified as:

(7) $$\frac{da}{dN}=C\Delta K_{\varepsilon }^{n}{\left(1-\frac{\Delta {K_{\varepsilon }}_{th}}{\Delta K_{\varepsilon }}\right)}^{p}$$

where *C*, *n*, and *p* are the fitting parameters and $\Delta K_{\varepsilon th}$ is the threshold strain intensity factor. Thus, the fatigue life can be calculated by integrating the fatigue crack growth rate equation, which can be expressed as follows:

(8) $$N=\int_{a_{i}}^{a_{c}}\frac{1}{C\Delta K_{\varepsilon }^{n}{\left(1-\frac{\Delta {K_{\varepsilon }}_{th}}{\Delta K_{\varepsilon }}\right)}^{p}}da$$

where *a_i_* is the initial crack size and *a_c_* is the critical crack size at failure. When the crack length is approaching *a_c_*, the crack growth rate is very fast. Some previous studies claim that the fatigue life is not sensitive to the value of *a_c_* [5,43] (in this study, it is determined by *K_c_* ($K_{c}=\gamma \sigma \sqrt{\pi a_{c}}$)). It can be observed from Equation (8) that the initial crack length plays an important role in the fatigue life evaluation. Since the real small crack growth is irregular and hard to describe accurately, the EIFS concept is proposed to estimate the small crack growth life [5]. As shown in Figure 5, the area S_1_ and S_0_ represent the fatigue life of the real crack, and the area S_2_ and S_0_ represent the life calculated by using EIFS. It is assumed that the area S_1_ is equal to the area S_2_. In other words, the long crack growth model and the EIFS are used to approximate the real small crack growth.

**Figure 6:**
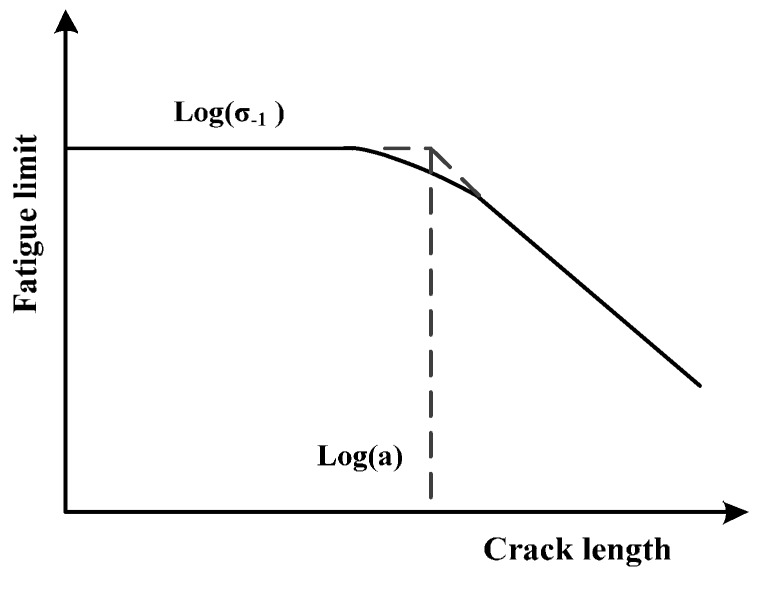
KT (Kitagawa-Takahashi) diagram.

In Figure 6, the Kitagawa-Takahashi diagram (KT diagram) shows the stress level decreases with the increase of the crack length until the stress reaches the fatigue limit [44].

El Haddad [45] et al. proposed an equation combining the threshold stress intensity factor range $\Delta K_{th}$ and the fatigue limit $\Delta \sigma_{-1}$

(9) $$\Delta K_{th}=\gamma \Delta \sigma_{-1}\sqrt{\pi a}$$

in which the crack length (a) is considered to be the EIFS. Under this condition, the stress and strain is almost linearly related, for the stress range is quite small. Thus, the EIFS can be calculated by the strain intensity factor and the value can be obtained by the threshold strain intensity factor and the fatigue limit strain:

(10) $$\Delta {K_{\varepsilon }}_{th}=\gamma \Delta \varepsilon_{-1}\sqrt{\pi a}$$

where $\Delta \varepsilon_{-1}$ is the fatigue limit strain. The threshold strain intensity factor and fatigue limit strain are defined as the threshold values, below which the fatigue life is infinite in theory. 

**Figure 7:**
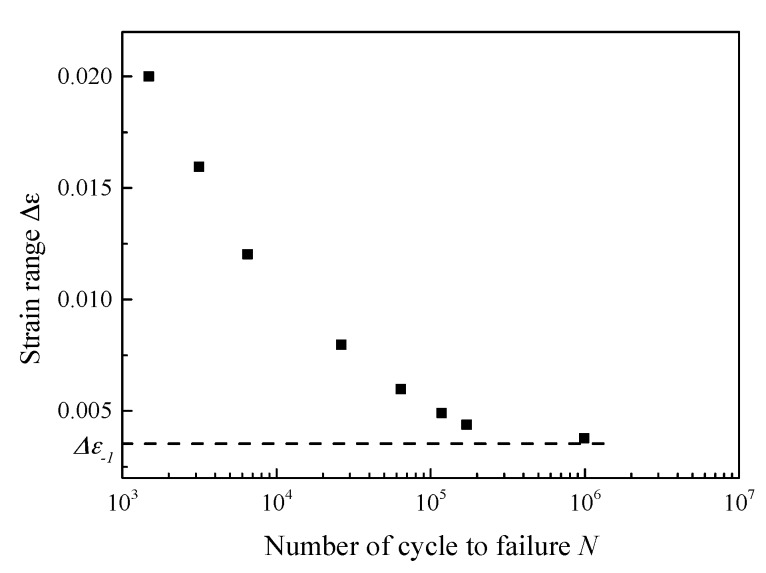
Fatigue life curve of 316 stainless steel.

Then, the EIFS is calculated by solving the Equation (10) numerically. Note that *γ* is also the function of the crack length. $\Delta {K_{\varepsilon }}_{th}$ is determined by back-extrapolation (the detailed procedure can be found in Reference [5]). $\Delta \varepsilon_{-1}$ is obtained by analyzing the asymptotic properties of the *ε-N* curve (as shown in Figure 7). Then, the fatigue life can be estimated as:

(11) $$N=\int_{EIFS}^{a_{c}}\frac{1}{C\Delta K_{\varepsilon }^{n}{\left(1-\frac{\Delta {K_{\varepsilon }}_{th}}{\Delta K_{\varepsilon }}\right)}^{p}}da$$

## 3. Validation and Comparison

### 3.1. Life Prediction for the 316 Austenitic Stainless Steel Specimens

The experimental data of cylindrical specimens in 316 austenitic stainless steel and extruded AZ31 magnesium alloy are employed to validate the proposed method under the fully reversed loading with the frequencies 0.2~2 Hz and 30 Hz respectively.

The main composition and the mechanical properties are now shown in Table 1 and Table 3.

**Figure 8:**
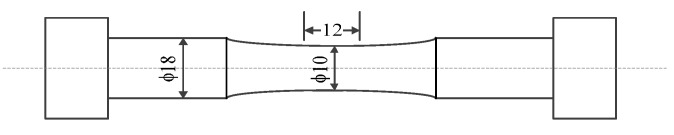
The configuration of the 316 stainless steel specimen.

The configuration of the 316 stainless steel specimen is shown in Figure 8. The diameter of the middle part is 10 mm, and the gauge length is 12 mm.

**Figure 9:**
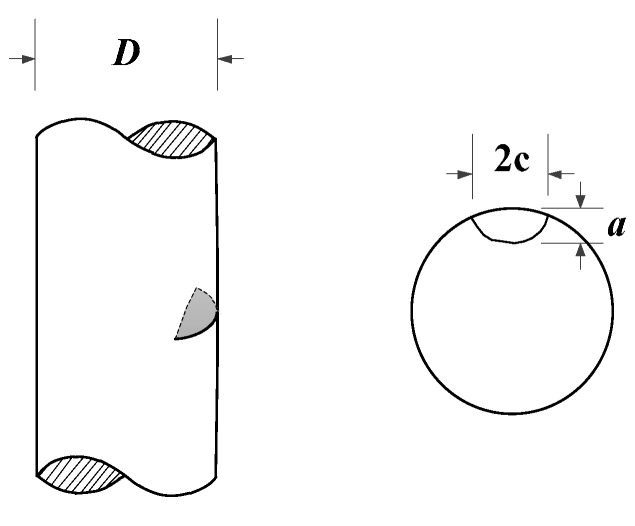
Schematic representation of surface crack in the specimen.

The surface crack is shown in Figure 9: *a* represents the crack depth, and 2*c* is the length of the surface crack. The ratio *a*/*c* is assumed to equal to one [37]. The geometry correction factor *γ* of the surface crack in a cylindrical specimen can be expressed as Equation (12) [46,47,48,49]

(12) $$\gamma =GG_{1}$$

where

(13) $$\begin{array}{l} G=0.92\frac{2}{\pi }\sec\beta {\left(\frac{\tan\beta }{\beta }\right)}^{0.5}\\ \beta =\frac{2}{\pi }\left(\frac{a}{D}\right)\\ G_{1}=0.752+1.286\beta +0.37A^{3}\\ A=1-\sin\beta \end{array}$$

**Figure 10:**
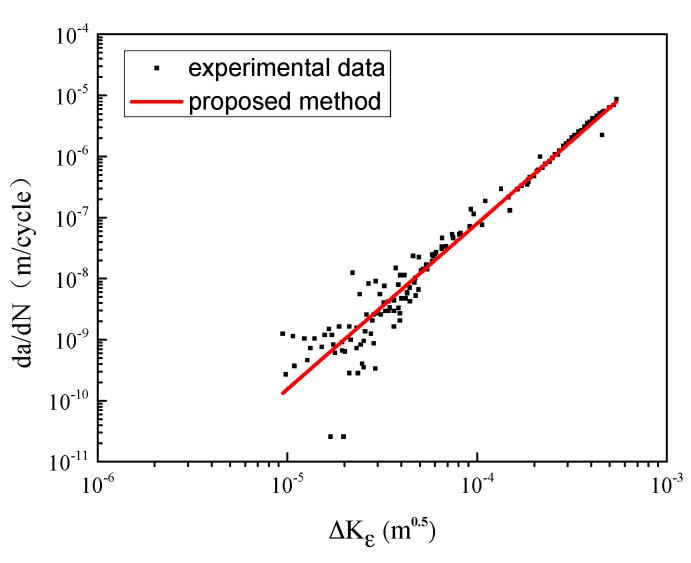
Crack growth rate of 316 stainless steel.

**Figure 11:**
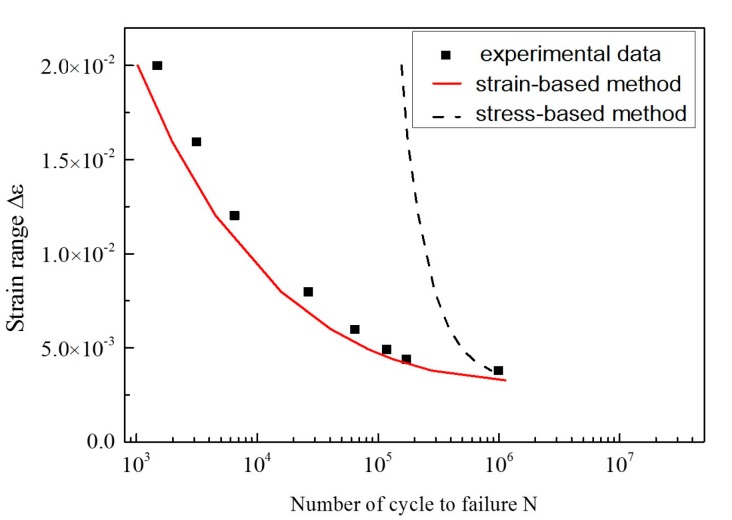
Predicted fatigue life of 316 stainless steel.

The fatigue limit strain $\Delta \varepsilon_{-1}$ is estimated as 0.321% by analyzing the *ε-N* curve in Figure 7 (the crack growth rate can be calculated based on the strain intensity factor). As is diagrammed in Figure 10, the calibrated coefficients of the fitting curve are *C* = 5689, *n* = 2.72. The fitting parameter *p* is assumed to be 1.5 [42]. The EIFS is evaluated to be 5.5696 μm by Equation (10). Then, the fatigue life can be assessed by using the proposed model. As shown in Figure 11, the dots indicate the experimental data, and the red line represents the model prediction. The calculation of the SIF-based method is also plotted for comparison, where the Equation (6) serves as the crack growth function and its calibrated coefficients are *C*_2_ = 5689, *n*_2_ = 2.72, *p* = 1.5; the EIFS is 4.8746 μm. A more detailed procedure of the SIF-based algorithm can be referred to Reference [43,50]. The predicted fatigue life is illustrated as the dash line in Figure 11. It is clear that the result of the strain-intensity-factor-based method matches the testing data very well. However, the predicted fatigue life based on the SIF has a large difference compared with the experimental data.

### 3.2. Life Prediction for Extruded AZ31 Magnesium Alloy Specimens

The main composition and the mechanical properties of extruded AZ31 magnesium alloy are now shown in Table 4 and Table 5 respectively [51].

The yield strength is 200 MPa; Young’s modulus is 45 GPa. The cyclic strength coefficient and cyclic strain hardening coefficient are 1976 and 0.34 respectively.

**Figure 12:**
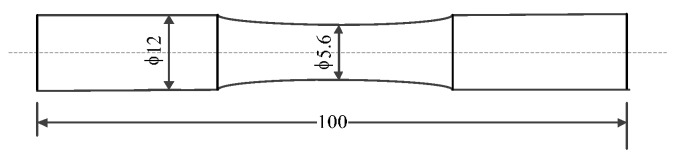
The dimensions of the AZ31 magnesium alloy specimen.

The dimensions of the AZ31 magnesium alloy specimen are shown in Figure 12. The diameter of the middle part is 5.6 mm.

**Figure 13:**
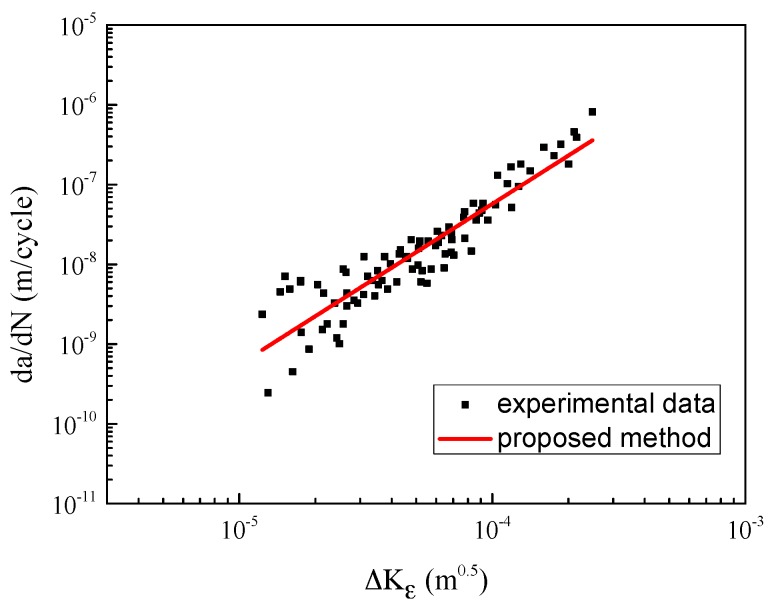
Crack growth rate of AZ31 magnesium alloy.

**Figure 14:**
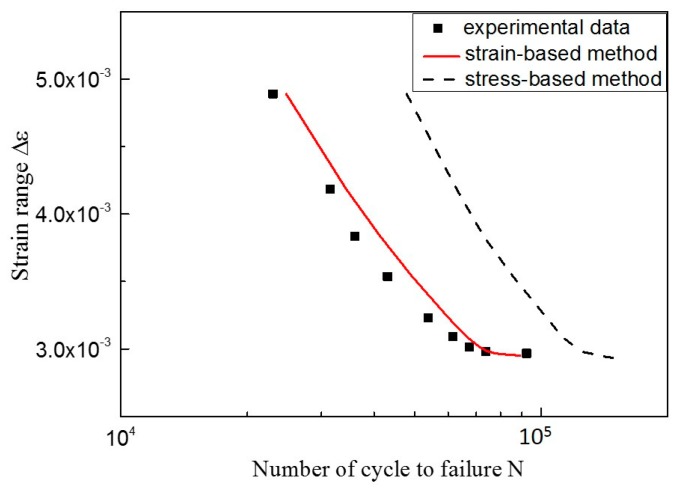
Predicted fatigue life of AZ31 magnesium alloy.

Similarly, *a*/*c* is assumed to equal to one; the fatigue limit strain $\Delta \varepsilon_{-1}$ is 0.27% [51]. The fitting curve of the crack growth rate is illustrated in Figure 13 and the calibrated coefficients are *C* = 8.4, *n* = 1.99; the fitting parameter p is assumed to be 0.5 [42]; and the EIFS is 1.3613 μm. Next, the prediction curve and experimental data are plotted in Figure 14, where the black dots represent the experimental data and the red line represents the predicted fatigue life by our proposed method. As shown, the predicted curve is in a better agreement with the experimental data. Similarly, the result of the SIF-based method is also shown as the dash line, where the calibrated coefficients are *C*_2_ = 1.995 × 10^−9^, *n*_2_ = 2.195, *p* = 0.5; the EIFS is 3.3459 μm. The strain-intensity-factor-based method fits the experimental data much better than the SIF-based method.

## 4. Conclusions

(1) A strain-intensity-factor-based crack growth model is proposed in this paper and a theoretical analysis has been provided to explicate the strain intensity factor as a driving parameter under the symmetrical cyclic loading. The experimental data in 316 austenitic stainless steel and AZ31 magnesium alloy are used for model validation and good agreements are observed. The slight differences between our model predictions and the experimental data are probably induced by the fitting errors of the calibrated coefficients. Additionally, the SIF-based method is also adopted for comparison. Next, some conclusions are summarized: A transformation algorithm between the SIF and the strain intensity factor is developed and validated by the fatigue testing data of 316 austenitic stainless steel and AZ31 magnesium alloy. It is clear that the dispersity of the crack growth rate vs. the strain intensity factor is lower than that vs. the SIF for different load ranges, which reveals that the strain intensity factor could be a better parameter than the stress intensity factor under the fully reversed load condition.
(2) Based on the strain intensity factor, a fatigue life prediction method is developed, in which the modified EIFS is employed. Then, the SIF-based method and experimental data are used in comparison with our model. It is clear that our proposed method matches the testing data much better than the SIF-based one.
(3) The current study is performed under the fully reversed loading and for only two materials. In the future, the research will be extended to the asymmetric loading condition and other mental materials.

## Figures and Tables

**Table 1:** Mechanical properties used in the finite element (FE) analysis.

0.2% Proof Strength/Yield Stress	Young’s Modulus	Poisson’s Ratio	Cyclic Strength Coefficient	Cyclic Strain Hardening Coefficient	Applied Loading
297 MPa	202.5 GPa	0.3	691	0.154	200 MPa

**Table 2:** Comparison between the different constitutive relationships.

Constitutive Relationships	Linear Elastic and Perfect Plastic	Nonlinear Cyclic Stress-Strain Relationship	Relative Difference
Remote Strain (%)	0.098	0.131	25.2%
Maximum Local Strain at the Crack Tip	0.1431	0.2100	31.9%

**Table 3:** Chemical composition of 316 stainless steel (wt %).

C	Mn	P	S	Si	Cr	Mo	Ni	Fe
0.06	1.30	0.031	0.027	0.50	16.94	2.02	10.18	Bal.

**Table 4:** Chemical composition of extruded AZ31 magnesium alloy (wt %).

Al	Zn	Mn	Fe	Ni	Cu	Si	Ca	Mg
2.98	0.97	0.004	0.007	0.005	0.002	0.02	0.05	Bal.

**Table 5:** Mechanical properties of extruded AZ31 magnesium alloy [31].

Yield Strength	Young’s Modulus	Cyclic Strength Coefficient	Cyclic Strain Hardening Coefficient
200 MPa	45 GPa	1976	0.34

## Nomenclature

a	Crack size/length
$a_{c}$	Critical crack size/length
$a_{i}$	Initial crack size
E	Young’s modulus
EIFS	Equivalent initial flaw size
*LEFM*	Linear elastic fracture mechanics
Δ*K*	Stress intensity factor range
*K_c_*	Critical stress intensity factor
*K_max_*	Maximum stress intensity factor
Δ*K_th_*	Threshold stress intensity factor range
Δ*K_ε_*	Strain intensity factor range
Δ*K_εth_*	Threshold strain intensity factor range
$K^{\prime }$	Cyclic strength coefficient
$n^{\prime }$	Cyclic strain hardening coefficient
R	Stress ratio
*SIF*	Stress intensity factor
γ	The geometrical coefficient
*ε*	Strain
*ε*_−1_	Fatigue limit strain
*ε_e_*	Elastic strain
*ε_p_*	Plastic strain
*σ*	Stress
*σ*_−1_	Fatigue limit
